# Identifying specific non-attending groups in breast cancer screening - population-based registry study of participation and socio-demography

**DOI:** 10.1186/1471-2407-12-518

**Published:** 2012-11-14

**Authors:** Line Flytkjær Jensen, Anette Fischer Pedersen, Berit Andersen, Peter Vedsted

**Affiliations:** 1 The Research Unit for General Practice, Department of Public Health, Aarhus University, Bartholins Allé 2, Aarhus C, 8000, Denmark; 2 Section for General Practice, Department of Public Health, Aarhus University, Bartholins Allé 2, Aarhus C, 8000, Denmark; 3 The Research Centre for Cancer Diagnosis in Primary Care (CaP), Aarhus University, Bartholins Allé 2, Aarhus, C 8000, Denmark; 4 Department for Public Health Programs, Regional Hospital of Randers, Skovlyvej 1, Randers, 8930, Denmark

**Keywords:** Screening, Mammography, Non-participation, Socio-demography, Inequality

## Abstract

**Background:**

A population-based breast cancer screening programme was implemented in the Central Denmark Region in 2008–09. The objective of this registry-based study was to examine the association between socio-demographic characteristics and screening participation and to examine whether the group of non-participants can be regarded as a homogeneous group of women.

**Method:**

Participation status was obtained from a regional database for all women invited to the first screening round in the Central Denmark Region in 2008–2009 (n=149,234). Participation data was linked to registries containing socio-demographic information. Distance to screening site was calculated using ArcGIS. Participation was divided into ‘participants’ and ‘non-participants’, and non-participants were further stratified into ‘active non-participants’ and ‘passive non-participants’ based on whether the woman called and cancelled her participation or was a ‘no-show’.

**Results:**

The screening participation rate was 78.9%. In multivariate analyses, non-participation was associated with older age, immigrant status, low OECD-adjusted household income, high and low level education compared with middle level education, unemployment, being unmarried, distance to screening site >20 km, being a tenant and no access to a vehicle. Active and passive non-participants comprised two distinct groups with different socio-demographic characteristics, with passive non-participants being more socially deprived compared with active non-participants.

**Conclusion:**

Non-participation was associated with low social status e.g. low income, unemployment, no access to vehicle and status as tenant. Non-participants were also more likely than participants to be older, single, and of non-Danish origin. Compared to active non-participants, passive non-participants were characterized by e.g. lower income and lower educational level. Different interventions might be warranted to increase participation in the two non-participant groups.

## Background

Breast cancer screening has been proven to reduce breast cancer mortality [1,2]. Although recent years have been characterized by discussions on the effectiveness of the programme [3], several health authorities, including the Danish, recommend regular screening for breast cancer. By law, all Danish women aged 50–69 must be offered biennial free-of-charge breast cancer screening by the region where the woman is resident [4].

A high participation rate across the target group is essential for the screening programme to be effective [4]. Therefore, non-participation becomes an important issue for health care planners. The associations between socio-demographic factors and non-participation have been studied for many years, and in most studies non-participation is associated with older age, being an immigrant, unemployment, being unmarried, low income, and being a tenant [5-12]. Mixed results have been found in relation to educational level [7,9,12,13] and distance to screening site [8,14-16].

Many studies have included only a limited number of socio-demographic factors and have not been able to draw a full picture of the often complex associations between socio-demography and non-participation. Except from a few studies [5-8], the studies conducted so far have typically relied on self-reported data on mammography uptake or socio-demographic data [9-13,16,17], and only two studies, based on the same study population, have been conducted in a Danish context [7,18]. Research in Denmark is of particular interest and importance due to a high relative breast cancer mortality compared to comparable countries [19].

Apart from Aro *et al.,*[17], no studies have to our knowledge explored to what extent the non-participant group comprises a homogeneous group of women. Women may have different motivations and reasons for non-participation. For instance, a woman might decide not to participate because she believes the harms of screening exceed the benefits. Contrary, other women might lack the resources to participate or even decide whether to participate or not. The ability to distinguish between the different groups of non-participants will therefore be important in order to perform group-specific interventions.

Using Danish registries, it was possible to conduct a large-scale study including all women invited to the first screening round in the Central Denmark Region and also to include many registry-based variables and thus not rely on self-report. The objective of this registry-based study was to examine the association between socio-demographic characteristics and screening participation and to examine whether the group of non-participants can be regarded as a homogeneous group of women.

## Methods

The first screening round took place from 28 February 2008 to 31 December 2009 where women were offered a pre-booked mammogram appointment at one of the region’s six screening sites. Appointment and site could be changed by the women who could also decline to participate. Non-participants received no reminders. A population-based study was performed including all women (aged 50–69) invited to the first screening round in the Central Denmark Region (n = 149,234).

According to Danish Legislation and the Central Denmark Region Committees on Biomedical Research Ethics (j.no.: 181/2011) the study did not need formal ethical approval, as it was based on registry data. The project was approved by the Danish Data Protection Agency (j.no.: 2009-41-3471 and j.no.: 1-16-02-31-11).

### Inclusion and exclusion criteria

All women invited to the first screening round were eligible for inclusion. For the purpose of this study, however, the following groups of women were excluded: Women who had died in the time between sending out the invitations and the scheduled day of the mammography (screening date) (n = 110), and women who had migrated from the Central Denmark Region before the screening date (n = 123). Women registered in the Danish Cancer Registry with a breast cancer diagnosis prior to the screening date were excluded (n = 4,646) because the majority of them were enrolled in a post-cancer follow-up period and hence did not participate in the organised screening programme. Finally, 91 women were excluded since they were listed with a general practitioner (GP) located in the Capital Region of Denmark. This left data on 144,264 (96.7%) women for analysis.

### Registries and variables

Data on participation was collected from a regional database containing administrative information. Participation was categorised as ‘participants’ or ‘non-participants’ based on whether the women participated in the first screening round or not. Non-participants were further divided into ’active non-participants’ (ANPs), defined as women who actively called and declined participation, and ‘passive non-participants’ (PNPs), defined as women who stayed away without cancelling or rescheduling the appointment (‘no-shows’).

Data on socio-demographic variables was obtained from ‘The Danish Integrated Database for Labour Market Research’ (IDA) run by Statistics Denmark [20], which has been updated annually since 1980. The following variables from IDA were included: Educational level classified according to UNESCO classification as low (≤10 years), middle (11–15 years), and higher education (>15 years). Labour positions were classified as: 1. employed, 2. self-employed and chief executive, 3. unemployed or receiving supplementary benefits other than social welfare, 4. retired women, 5. social welfare recipients, and 6. others. Marital status was classified as married, living in a registered partnership, cohabitating, or being single. Ethnicity was categorised as Danish, immigrants from western countries, and immigrants from non-western countries, according to Statistics Denmark’s definition of developed countries [21]. Residential ownership was divided into residence-owners and tenants. ‘OECD-adjusted household income’ in the year prior to mammography, adjusted for number of persons in the household [22], was used as an income measure. Based on tertiles and rounded off to the nearest 100 Euros, OECD-adjusted household income was categorised as: low (1st tertile, ≤34,600 Euros ), middle (2nd tertile, >34,600 to ≤53,200 Euros ), and high (3rd tertile, >53,200 Euros). From the National Vehicle Registry, information on the household’s access to a vehicle was obtained and divided into access or no access. From The Danish Cancer Registry [23], all registered cancer diagnoses prior to the screening date were obtained, and all women with a prior breast cancer diagnosis were identified and excluded. The remaining women were divided into either ‘no registrations of a cancer diagnosis’ or ‘registered with one or more cancer diagnoses (excluding breast cancer) prior to the screening date’. Women diagnosed with cancer prior to the age of 14 were categorized as ‘no registered cancers’ (n = 62).

Travel distance to screening site was calculated according to the Danish road network using ArcGIS Network Analyst (version 10.0) [24]. Geographical coordinates were used to locate each woman’s residence on the date of her screening appointment and the coordinates were obtained from the Centralised Civil Register. It was possible to calculate the shortest driving route in km for 137,417 of the women (95.3%). Travel distance was categorised into: 1) 0–20 km, 2) >20-40 km, 3) >40-60 km, and 4) >60 km.

In Denmark 98% of citizens are listed with a general practice, which they must contact for medical advice [25]. From the ‘Patient Registry’, data on which general practice the woman was listed with at the screening date was obtained.

All registries could be linked by the women’s unique civil registration number (CRN). A woman’s socio-demographic position was described the year prior to the booking date. This means that for a women being assigned to screening in e.g. 2009, socio-demographic registries updated at the end of 2008 were used. Missing information on the registry-based variables ranged from 0% for the variables ‘age’ and ‘participation’ to 1.8% for data on ‘residential ownership’.

### Analysis

Generalised linear models (GLM) with log link and the Bernoulli family regression models [26,27] were used to quantify the association between socio-demographic factors and screening participation. Restricting the analyses to the group of non-participants (n=30,453), the same method was applied to investigate whether ANPs and PNPs differed in terms of socio-demographic factors.

Initially, to check for multicollinearity between independent variables the mean Variance Inflation Factor (VIF) was calculated. Values above 10 indicate multicollinarity [28]. Following, unadjusted analyses were done with each independent variable. A multiadjusted model was also done, adjusting for statistically significant variables (p <0.01) from the unadjusted analysis. Prevalence ratios (PR) with 95% confidence intervals (CI) were used as association measures using robust variance estimates to allow for clustering of patients by general practice in both unadjusted and adjusted models [29]. Due to a high prevalence of the outcome measure (more than 20% non-participants), odds ratios tend to overestimate the association [26,27]. Age was calculated on the date of the screening appointment using the women’s CRN containing date and year of birth. Statistical analyses were conducted using STATA version 12.

## Results

### Sample characteristics

The participation rate in the first screening round was 78.9% for the final sample. Mean age for participants was 59.3 years and 60.0 years for non-participants. Table 1 shows the distribution of socio-demographic variables in terms of participation. Participants and non-participants were statistically significantly different (chi^2^ p <0.01) in all socio-demographic groups. Table 1 also depicts the distribution of PNPs and ANPs which was also significantly different in all groups (chi^2^ p <0.01). Mean age for PNPs was 59.4 years and 60.7 years for ANPs.

**Table 1:** Distribution of socio-demographic characteristics for participants (n=113,811), non-participants (n=30,453) and non-participants divided into active (n=14,529) and passive (n=15,924)

**Variable**	**Participants n= 113,811**		**Non-participants (ANP+PNP) n=30,453**		**Active non-participants (ANP) n= 14,529**		**Passive non-participants (PNP) n=15,924**	
	**n**	**% column**	**n**	**% column**	**n**	**% column**	**n**	**% column**
**Age (years)**								
50-54	30,965	27.2	7,536	24.7	3,022	20.8	4,514	28.4
55-59	30,722	27.0	7,580	24.9	3,396	23.4	4,184	26.3
60-64	30,532	26.8	7,998	26.3	4,139	28.5	3,859	24.2
65+	21,592	19.0	7,339	24.1	3,972	27.3	3,367	21.1
**Ethnicity**								
Danish	110,018	96.7	28,201	92.7	13,967	96,2	14,234	89,5
Western immigrants	2,024	1.8	918	3.0	341	2,3	577	3,6
Non-western immigrants	1,749	1.5	1,306	4.3	217	1,5	1,089	6,8
**Marital status**								
Married	80,748	71.0	16,208	53.3	8,723	60.1	7,485	47.1
Registered partnership	96	0.1	46	0.2	25	0.2	21	0.1
Cohabitating	7,746	6.8	2,230	7.3	871	6.0	1,359	8.6
Single	25,183	22.1	11,924	39.2	4,900	33.7	7,024	44.2
**Occupation**								
Employed	63,169	55.5	12,270	40.4	5,973	41.1	6,297	39.6
Self-employed/chief executive	4,536	4.0	1,283	4.2	632	4.4	651	4.1
Unemployed/benefits*	14,103	12.4	6,817	22.4	2,549	17.6	4,268	26.8
Retired women	28,596	25.1	8,275	27.2	4,738	32.6	3,537	22.3
Social welfare recipients	609	0.5	531	1.8	72	0.5	459	2.9
Others	2,760	2.4	1,228	4.0	551	3.8	677	4.3
**Education (years)**								
≤ 10	39,214	34.9	12,651	42.8	5,403	37.9	7,248	47.4
11-15	47,661	42.4	10,624	35.9	5,601	39.2	5,023	32.9
>15	25,549	22.7	6,286	21.3	3,272	22.9	3,014	19.7
**OECD-adjusted household income†**								
Low tertile	33,484	29.4	14,476	47.6	6,056	41.7	8,420	53.0
Middle tertile	39,255	34.5	8,880	29.2	4,495	30.9	4,385	27.6
High tertile	41,034	36.1	7,048	23.2	3,964	27.3	3,084	19.4
**Access to vehicle**								
Yes	99,597	87.5	21,302	70.0	11,085	76.4	10,217	64.3
No	14,176	12.5	9,106	30.0	3,434	23.6	5,672	35.7
**Residential ownership**								
House owners	86,309	77.1	17,978	60.5	9,704	68.3	8,274	53,3
Tenants	25,648	22.9	11,751	39.5	4,511	31.7	7,240	46.7
**Kilometres to screening site**								
0-20 km	54,989	50.7	13,622	46.9	6,079	44.0	7,543	49.6
>20-40 km	32,535	30.0	8,599	29.6	4,260	30.9	4,339	28.5
>40-60 km	17,327	16.0	5,604	19.3	2,832	20.5	2,772	18.2
>60 km	3,545	3.3	1,198	4.1	633	4.6	565	3.7
**Previous cancer diagnoses**								
No	99,585	87.5	26,470	86.9	12,431	85.6	14,039	88.2
Yes	14,226	12.5	3,983	13.1	2,098	14.4	1,885	11.8

Numbers vary due to missing data.

* state benefits in relation to sickness, education, leave benefits, disability pension, and student grants.

†: see method.

### Associations between socio-demography and participation

Table 2 presents unadjusted and adjusted associations between participation and socio-demographic factors. No multicollinearity was observed between any of the independent variables with all VIF values ranging between 1.00-1.51.

**Table 2:** Unadjusted and adjusted prevalence ratios (PR), with 95% confidence intervals (CI) for associations between socio-demographic variables and screening non-participation

	**Unadjusted**	**Adjusted model**
**Age (years)**		
50-54	1 (ref)	1 (ref)
55-59	1.01 (0.98-1.04)	1.01 (0.98-1.04)
60-64	**1.06 (1.03-1.09)**	1.02 (0.98-1.05)
65+	**1.30 (1.26-1.34)**	**1.23 (1.17-1.29)**
**Ethnicity**		
Danish	1 (ref)	1 (ref)
Western immigrants	**1.53 (1.45-1.61)**	**1.27 (1.19-1.36)**
Non-western immigrants	**2.10 (1.98-2.22)**	**1.38 (1.30-1.46)**
**Marital status**		
Married	1 (ref)	1 (ref)
Registered partnership	**1.94 (1.47-2.56)**	**1.76 (1.31-2.38)**
Cohabitating	**1.34 (1.28-1.39)**	**1.27 (1.22-1.33)**
Single	**1.92 (1.88-1.97)**	**1.30 (1.27-1.34)**
**Education**		
≤10	**1.34 (1.30-1.37)**	**1.10 (1.08-1.13)**
11-15	1 (ref)	1 (ref)
>15	**1.08 (1.05-1.12)**	**1.15 (1.11-1.18)**
**Previous cancer diagnoses**		
No	1 (ref)	1 (ref)
Yes	**1.04 (1.01-1.07)**	1.02 (0.99-1.05)
**Occupation**		
Employed	1 (ref)	1 (ref)
Self-employed and chief executive	**1.36 (1.28-1.44)**	**1.33 (1.26-1.41)**
Unemployed/receiving benefits*	**2.00 (1.94-2.07)**	**1.35 (1.30-1.39)**
Retired women	**1.38 (1.34-1.42)**	1.00 (0.96-1.05)
Social welfare recipients	**2.86 (2.66-3.08)**	**1.54 (1.42-1.67)**
Others	**1.89 (1.79-2.00)**	**1.51 (1.42-1.60)**
**OECD-adjusted household income†**		
High tertile	1 (ref)	1 (ref)
Middle tertile	**1.26 (1.21-1.30)**	**1.11 (1.07-1.16)**
Low tertile	**2.06 (1.99-2.13)**	**1.29 (1.23-1.35)**
**Residential ownership**		
House owners	1 (ref)	1 (ref)
Tenants	**1.82 (1.77-1.87)**	**1.17 (1.14-1.21)**
**Kilometres to screening site**		
0-20 km	1 (ref)	1 (ref)
>20-40 km	1.05 (1.00-1.11)	**1.16 (1.10-1.21)**
>40-60 km	**1.23 (1.15-1.32)**	**1.32 (1.24-1.41)**
>60 km	**1.27 (1.15-1.40)**	**1.36 (1.24-1.49)**
**Access to vehicle**		
Yes	1 (ref)	1 (ref)
No	**2.22 (2.16-2.28)**	**1.46 (1.42-1.50)**

N =144,264 (non-participants: N=30,453, participants: N=113,811).

* State benefits in relation to sickness, education, leave benefits, disability retirement, and student grants.

†: see method.

Adjusted model: Adjusted for age, ethnicity, marital status, education, previous cancer diagnoses, occupation, OECD-adjusted household income, residential ownership, distance to screening unit and access to vehicle.

For most variables in the unadjusted model, statistically significant associations with non-participation were found. Especially women who were immigrants, older, unmarried, tenants, outside the workforce, had a low-level or a high-level education, a low income, and women with no access to a vehicle were more likely not to participate (Table 2).

In the fully adjusted model, most of the estimates decreased compared with the unadjusted analyses. The largest decrease was observed for ‘non-western immigrants’, ‘low income’, and single women. Having a previous cancer diagnosis was no longer statistically significantly associated with participation. Also, only belonging to the oldest age group was now statistically significantly associated with non-participation compared with the youngest group, and retired women were no longer statistically significantly more likely not to participate than employed women. An increase was observed among highly educated women in the adjusted model, indicating a 15% increased risk of non-participation compared with women with a middle level education. The association between distance and participation also increased compared with the unadjusted analysis and all categories in this variable were statistically significantly associated to participation in the adjusted model (Table 2).

### Active and passive non-participants

The association between active and passive non-participants is presented in Table 3. In terms of multicollinearity, all VIF values were between 1.00-1.42. The unadjusted analysis showed that PNPs were more likely to be immigrants, unmarried, younger, social welfare recipients, unemployed, having a low-level education, a low household income, being tenants, having no access to a vehicle, and having a shorter distance to screening site than ANPs. ANPs were more likely to have had a previous cancer diagnosis.

**Table 3:** Unadjusted and adjusted prevalence ratios (PR), with 95% confidence intervals (CI) for association between socio-demographic factors and being passive non-participants (PNP)

	**Unadjusted**	**Adjusted model**
**Age (years)**		
50-54	1 (ref)	1 (ref)
55-59	**0.92 (0.90-0.95)**	**0.93 (0.91-0.96)**
60-64	**0.81 (0.78-0.83)**	**0.85 (0.82-0.88)**
65+	**0.77 (0.74-0.79)**	**0.88 (0.84-0.92)**
**Ethnicity**		
Danish	1 (ref)	1 (ref)
Western immigrants	**1.25 (1.19-1.31)**	**1.22 (1.15-1.29)**
Non-western immigrants	**1.65 (1.59-1.71)**	**1.39 (1.34-1.45)**
**Marital status**		
Married	1 (ref)	1 (ref)
Registered partnership	0.99 (0.70-1.39)	0.91 (0.64-1.30)
Cohabitating	**1.32 (1.27-1.38)**	**1.23 (1.18-1.28)**
Single	**1.28 (1.24-1.31)**	**1.09 (1.06-1.12)**
**Education**		
≤10	**1.21 (1.18-1.25)**	**1.18 (1.15-1.21)**
11-15	1 (ref)	1 (ref)
>15	1.01 (0.98-1.05)	1.02 (0.99-1.06)
**Previous cancer diagnoses**		
No	1 (ref)	1 (ref)
Yes	**0.89 (0.86-0.93)**	**0.92 (0.89-0.95)**
**Occupation**		
Employed	1 (ref)	1 (ref)
Self-employed and chief executive	0.99 (0.93-1.05)	1.01 (0.95-1.08)
Unemployed/receiving benefits*	**1.22 (1.18-1.26)**	1.00 (0.97-1.03)
Retired women	**0.83 (0.81-0.86)**	**0.78 (0.74-0.81)**
Social welfare recipients	**1.68 (1.62-1.76)**	**1.14 (1.09-1.20)**
Others	**1.07 (1.01-1.14)**	0.95 (0.89-1.02)
**OECD-adjusted household income†**		
High tertile	1 (ref)	1 (ref)
Middle tertile	**1.13 (1.09-1.17)**	**1.08 (1.04-1.13)**
Low tertile	**1.33 (1.28-1.38)**	**1.22 (1.17-1.27)**
**Residential ownership**		
House owners	1 (ref)	1 (ref)
Tenants	**1.34 (1.30-1.37)**	**1.10 (1.07-1.14)**
**Kilometres to screening site**		
0-20 km	1 (ref)	1 (ref)
>20-40 km	0.91 (0.88-0.95)	**0.95 (0.92-0.99)**
>40-60 km	**0.89 (0.85-0.94)**	**0.93 (0.89-0.98)**
>60 km	**0.85 (0.78-0.93)**	**0.88 (0.82-0.95)**
**Access to vehicle**		
Yes	1 (ref)	1 (ref)
No	**1.30 (1.27-1.33)**	**1.06 (1.02-1.09)**

N =30,453 (passive non-participants: N=15,924, active non-participants: N=14,529).

* State benefits in relation to sickness, education, leave benefits, disability retirement, and student grants.

†: see method.

Adjusted model: Adjusted for age, ethnicity, marital status, education, previous cancer diagnoses, occupation, OECD-adjusted household income, residential ownership, distance to screening unit and access to vehicle.

In the fully adjusted model, all associations decreased or remained stable when compared with the unadjusted analyses. In relation to occupation, being unemployed and ‘other’ was no longer statistically significantly associated with non-participation. Contrary, all categories in ‘distance to screening unit’ were now statistically significantly associated to participation in the adjusted model. There was no statistically significantly association between the highest educated group and participation in neither the unadjusted or adjusted analysis (Table 3).

## Discussion

### Main findings

Approximately one in five women invited to the first screening round in the Central Denmark Region did not participate. This study indicates a social gradient in the participation as women outside the workforce, with a low income, from non-western countries, being unmarried, and not owning their home, and having no access to a vehicle were more likely not to participate. Nearly half of the non-participants had actively decided not to participate. When comparing passive non-participants (PNPs) with active non-participants (ANPs), the two groups differed distinctively from one another as PNPs were more likely to belong to the lowest social groups with e.g. low income and low education.

### Strengths and weaknesses

Linkage of Danish registries using the unique CRN made it possible to conduct a large-scale study that included several complete variables with a high validity [30]. This population-based study included the entire population of invited women and was based on the first screening round. This makes this population favourable to study because no women were excluded on the grounds that they had previously actively chosen not to take part in the programme. The study used data on screening uptake and socio-demography from regional and national registries and did not rely on self-report. This minimised selection and information bias and enhanced the validity and generalisability of the study. The large number of included women ensured high statistical precision which made it possible to include several variables in the analyses without losing precision. Further, the study was able to exclude very precisely women who died before the screening date, moved out of the region or had previously been diagnosed with breast cancer. Finally, this study is of special interest as it is one of the first studies in this field to further investigate the group of non-participants.

Some weaknesses of this study should also be mentioned. Socio-demographic variables from IDA are updated once annually. It was chosen to use the datasets describing the women’s socio-demographic characteristics the year prior to the screening date. However, socio-demographic variables such as marital status and occupation are dynamic and some women may e.g. have changed jobs during the study period which leads to misclassification. Further, the ’National Vehicle Registry’ did not include leased cars and business cars, and the true prevalence of people having access to a vehicle might therefore be underestimated.

The calculated distance to screening site using the Danish road network and the women’s residence gave exact estimates of the distances. However, the assumption that all women travelled from their home address may not be valid. Furthermore, 5.2% of the women rescheduled their appointment to another screening site which for some of the women may have been to a screening site near their workplace, but far away from their homes. However, excluding these women from the analysis did not alter the association markedly (data not shown).

Using Danish registries, various complete variables were possible to include in the study. However, residual confounding cannot be excluded, since other variables of importance may not have been included. E.g. family history of breast cancer and more psychologically related variables such as fear may be important in this context as well [31-33].

### Comparison of participants and non-participants

Corresponding to results from a Swedish [6] and a Danish [7] study, our study found that both women with a low- and high level education were less likely to participate compared with women with a middle-level education. The association between a low-level education and non-participation may to some extent be explained by a lower ability among women with a low-level education to obtain, process, and understand health information [34]. Conversely, it has been suggested that women of a high educational level are more likely to use private mammography services which could explain the association between high education and non-participation [5]. However, this does not seem to be the case in Denmark [35]. Another explanation could be that highly educated women have followed the ongoing debate regarding benefits and harms of screening and decided not to participate. Finally, one reason could be that highly educated women more often have increased occupational responsibilities, and a lack of time could be a barrier to mammography screening participation [36]. In this study this hypothesis is supported by the fact that the group of self-employed and chief executives was less likely to participate compared with employed women. However, the association between education and participation is clearly not established and one explanation might be that non-participants do not comprise a homogeneous group of women as demonstrated in this study.

In this study and others [5,6,9], being outside the workforce was associated with lower participation rates. This could be due to increased social interaction and support from colleagues [6], but it could also be an indication of a poor health status of women outside the workforce [6]. OECD-adjusted household income has been suggested to be a more reliable measure of economic status than personal income [5]. Lower OECD-adjusted household income was associated with higher risk of non-participation in this study which corresponds with results from other studies [5,10,12]. Although associations between various socio-economic variables and participation have been indicated in many subpopulations and different health care systems [6,8,12,14,37,38], this study indicated that even in a health care system with universal coverage and no payment for mammography service, social inequalities in participation exist.

Married women were more likely to be participants which is in line with the findings of other studies [5,7,8,11] and could be explained by increased support and decision aid from their significant others. As shown in many studies [7,8,10,11], although not in all [5,13], older age was associated with a higher risk of non-participation. As concluded by many other studies [5-8], immigrants were more likely not to participate. This was especially the case for women with a non-western background and can probably be explained by linguistic, cultural, and religious barriers [37,38]. Interventions towards immigrants might be of great value in terms of increasing this particular group's participation rate. Long distance to screening site was associated with a higher likelihood of non-participation. Studies from both Europe and USA do not agree whether distance to screening site matters in terms of participation [8,14-16]. Since this knowledge might be of great value for public health planners, the mechanisms of this association should be studied further.

### Comparison of ANPs and PNPs

To the authors’ knowledge, only one study has previously explored whether the group of non-participants comprises one group with the same characteristics [17]. This present study clearly shows that ANPs and PNPs comprise two different groups of women with different socio-demographic characteristics. When ANPs called and declined participation, one could put forward the hypothesis that ANPs had made a deliberate decision of not wishing to participate whereas PNPs might fail to turn up due to lack of personal or practical resources. This hypothesis is supported by the results of this study indicating that PNPs are more likely to be of a low social status with e.g. a low-level education and a low income. To reach these two groups of non-participants, different interventions might be needed since fundamental demographic and economic characteristics vary within the groups. Also the ethics of targeting ANPs must be considered, given the fact that they may already have thoroughly considered their decision not to participate. However, more studies are needed to identify specific reasons for non-participation among the two groups of non-participants.

## Conclusions

Despite the free-of-charge programme in Denmark, non-participation appeared to be determined by social disparities since non-participants were more likely to have low income, no access to vehicle, be unemployed, and not own their own home. Non-participants were also more likely to be older, single, and of non-Danish origin. Further, the group of non-participants comprised two different groups of women with different socio-demographic characteristics which clearly show the complexity of screening behaviour. Thus, passive non-participants were more likely to e.g. have low income and low educational attainment than active non-participants. Future interventions may profit from focusing on this social inequality and also from targeting the different groups of non-participants.

## Competing interests

The authors declare that they have no competing interests.

## Authors’ contributions

AFP, BA, and PV conceived the idea. LFJ, PV, AFP, and BA contributed with input and critical revision of the statistical analyses and content of the paper. LFJ was primarily responsible for the statistical analyses and for drafting the manuscript. All authors read and approved the final manuscript.

## Pre-publication history

The pre-publication history for this paper can be accessed here:

http://www.biomedcentral.com/1471-2407/12/518/prepub
